# Diagnosis Unreliability of ChatGPT for Journal Evaluation

**DOI:** 10.34172/apb.2024.020

**Published:** 2023-10-14

**Authors:** Mehdi Dadkhah, Marilyn H Oermann, Mihály Hegedüs, Raghu Raman, Lóránt Dénes Dávid

**Affiliations:** ^1^Amrita School of Engineering, Amrita Vishwa Vidyapeetham, Amritapuri, Kerala, India.; ^2^Technology Forecasting Department, SnowaTec Technology Center and Innovation Factory, Entekhab Industrial Group, Isfahan, Iran.; ^3^School of Nursing, Duke University, Durham, North Carolina, USA.; ^4^Tomori Pál College, Chamber of Hungarian Auditors, Budapest, Hungary.; ^5^Amrita School of Business, Amrita Vishwa Vidyapeetham, Amritapuri, Kerala, India.; ^6^Faculty of Economics and Business, John von Neumann University, Kecskemét, Hungary.; ^7^Institute of Rural Development and Sustainable Economy, Hungarian University of Agriculture and Life Sciences, Gödöllő, Hungary.

**Keywords:** Hijacked journals, Predatory journals, ChatGPT, Artificial intelligence, Language models, Research ethics

## Abstract

**Purpose:**

Academic and other researchers have limited tools with which to address the current proliferation of predatory and hijacked journals. These journals can have negative effects on science, research funding, and the dissemination of information. As most predatory and hijacked journals are not error free, this study used ChatGPT, an artificial intelligence (AI) technology tool, to conduct an evaluation of journal quality.

**Methods:**

Predatory and hijacked journals were analyzed for reliability using ChatGPT, and the reliability of result have been discussed.

**Results:**

It shows that ChatGPT is an unreliable tool for journal quality evaluation for both hijacked and predatory journals.

**Conclusion:**

To show how to address this gap, an early trial version of Journal Checker Chatbot has been developed and is discussed as an alternative chatbot that can assist researchers in detecting hijacked journals.

## Introduction

 Artificial intelligence (AI) technology refers to the development of data systems that allow machines to perform tasks generally associated with human intelligence such as reasoning and decision making.^1^ Its use is becoming ubiquitous, and along with the benefits it offers, there are risks. AI tools present a paradigm shift in academic writing in that they can be used by authors to improve the quality of their papers and by editors to speed up the paper inspection process (e.g., detect plagiarism)^2^ but also by dishonest researchers for purposes of academic misconduct. A popular AI-based tool is a chatbot entitled ChatGPT (GPT stands for generative pre-trained transformer). ChatGPT is a language model developed by OpenAI which can answer questions based on an immense set of data collected from the internet. Users can chat with ChatGPT and receive answers to questions about various fields of knowledge^3-5^; however, research shows that ChatGPT may provide invalid output and unreliable data.

 Additionally, ChatGPT can be used by paper mill companies to generate fake research papers.^6-10^ As ChatGPT becomes increasingly popular, more researchers may ask it to recommend journals or to evaluate the quality and credibility of particular journals to which they intend to send queries. It is imperative to understand whether ChatGPT can provide trustworthy answers to such requests.

## Literature review

 Historically, two types of questionable journals have plagued academic publishing: predatory and hijacked journals. Predatory journals publish papers without fair review and do not follow the essential publishing process to provide high quality literature.^11-15^ There exists a rich body of knowledge as well as criteria and online lists to help researchers identify potential predatory journals and publishers.^16-19^ Hijacked journals are fake websites that list reputable indexed journals but have no relation to authentic journals; the actual journals are mimicked in order to charge authors fees and publish papers without peer review.^20-22^ The extent of journal hijacking is not limited to a particular domain, and indexed hijacked journals have appeared in the Clarivate (previously known as Thomson Reuters) and other citation databases.^23,24^ Both predatory and hijacked journals can damage scientific integrity, propagate errors, decrease countries’ scientific ranking, waste university budgets, and hurt academic reputations.^25-29^

 Our literature search for ChatGPT use for journal quality evaluation did not provide rich information. Most of the available studies are focused mainly on the detection of ChatGPT-generated papers and the possibility that such papers could be published in predatory journals. There is only one research study about ChatGPT use for journal quality evaluation and concluded that ChatGPT provides accurate answer^5^ about the current status of art.^30^ However, as most of the currently available methods and tools for detecting predatory and hijacked journals are complex or have critical weaknesses.^17,22,31^ many researchers may tend to use ChatGPT to evaluate and recommend journals before submitting a paper.

## Reliability of ChatGPT for journal quality evaluation

 The use of ChatGPT is widespread, yet there exist no strong evidences regarding its reliability in evaluating journal authenticity. In order fill this gap, we shaped three datasets. The first dataset contained a list of all currently detected hijacked journals because existing lists of hijacked journals were not up-to-date.^32-34^ We used an updated (to July 8, 2023) online list of hijacked journals published by Retraction Watch Blog entitled “Hijacked Journal Checker”^35^ URL: https://retractionwatch.com/the-retraction-watch-hijacked-journal-checker/ for this dataset.

**Figure 1:**
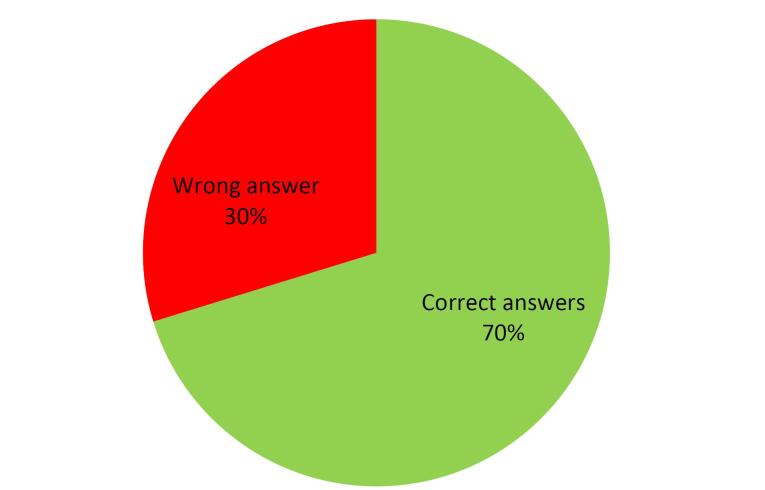
Percentage of correct answers regarding hijacked journals

**Figure 2:**
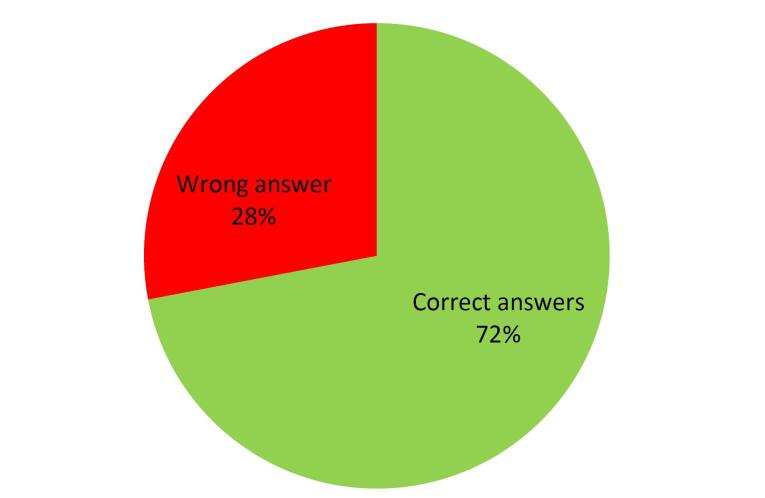
Percentage of correct answers regarding predatory journals

 The second dataset contained a list of 50 potential predatory publishers gathered from the https://beallslist.net by random sampling. The third dataset contained a list of 100 potential predatory journals gathered from https://beallslist.net by random sampling. There has been some criticism about the use of potential lists of predatory publishers and journals; however, we used these lists solely to examine ChatGPT’s ability to provide accurate information. We presented ChatGPT with the hijacked journals’ URLs and requested its opinion of the quality and authenticity of each URL. Additionally, we asked ChatGPT to provide its opinion on each journal/publisher on the of list of predatory journals and publishers (by use of exact name of journals and publishers). Results are shown in Figures 1 and 2.

 ChatGPT recommended about 30% of hijacked journals as reputable publications. For example, https://www.hfsp-journal.org is a hijacked version of an authentic journal, *Frontiers of Interdisciplinary Research in The Life Sciences* (https://www.hfsp.org/hfsp-news/alert-fake-journal-fraudulent-use-hfsp-name).^21^ When ChatGPT was asked, “Is https://www.hfsp-journal.org an authentic journal?” it responded:

 Yes, the HFSP Journal is an authentic journal. It is an open-access, peer-reviewed scientific journal that publishes original research articles, reviews, and perspectives in the fields of life sciences and related disciplines. The journal is published by the Human Frontier Science Program (HFSP) Organization, which is an international organization that supports frontier research in the life sciences. The HFSP Journal is indexed in several reputable databases, including PubMed Central, Web of Science, and Scopus, and its editorial board consists of experts in various fields of life sciences. The journal has a rigorous peer-review process in place to ensure the quality and validity of published research. Therefore, the HFSP Journal is a legitimate and reputable journal in the scientific community.

 When asked “Is https://www.hfsp-journal.org hijacked journal?” ChatGPT responded, “As far as I am aware, there is no evidence to suggest that the HFSP Journal is a hijacked journal”.

 Although ChatGPT demonstrated an ability to provide appropriate recommendations as well as accurate and rich information about potential predatory publishers, 28% of its responses were mistaken recommendations of predatory journals. Our findings were that ChatGPT is not error free in this respect and cannot be relied upon as a tool for journal quality evaluation. This means that ChatGPT did not use current literature well, because most of information about predatory and hijacked journals (plus their URLs) are freely available on internet from 2015 until now.

## The solution that we need: Journal Checker Chatbot

 Given the tendency of ChatGPT to mistakenly recommend questionable journals, we developed a trial limited “Journal Checker Chatbot,” using Botpress. It is a platform based on natural language recognition that provides a visual tool to set up a chatbot.^36,37^ There is also online version of Botpress that support easy publishing of chatbot in the web. This chatbot also uses OpenAI API to provide next-generation chatbots and more intelligence answers based on the provided dataset.^38,39^ For the purpose of this paper, only data pertaining to the identification and evaluation of authentic and hijacked journals were prepared to help the chatbot learn. The term “hijacked journals” was searched in Google Scholar (22 June 2022), and the full texts of all publications whose main theme pertained to hijacked journals were collected. In addition, an updated list of hijacked journals was collected and added to the dataset.

**Figure 3:**
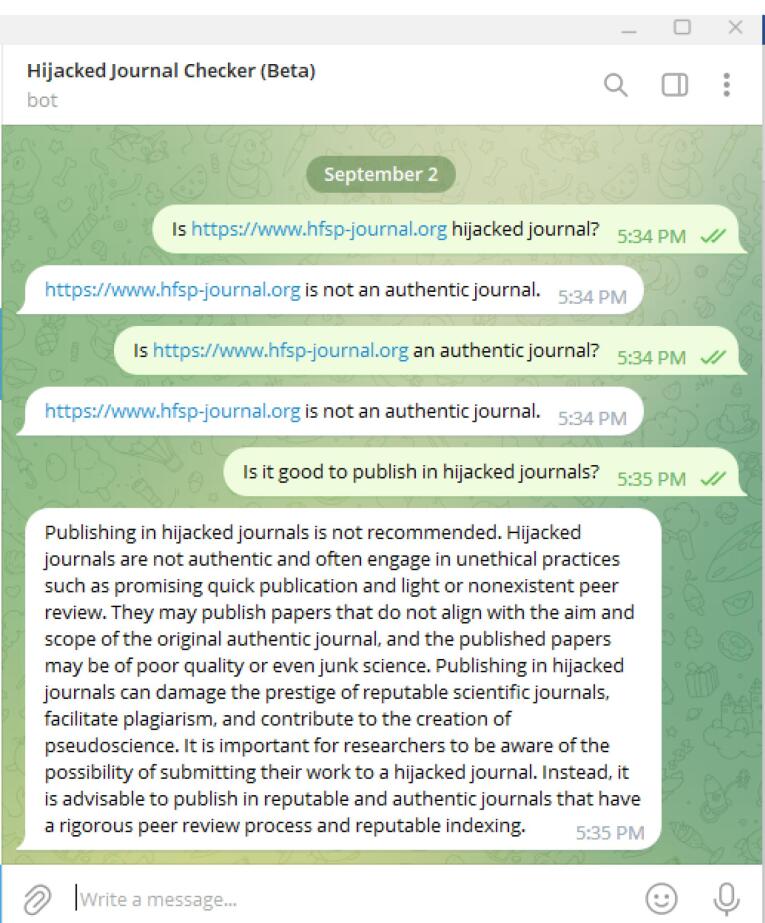
Screenshots from Journal Checker Chatbot

 The developed chatbot is accessible online via this address: https://t.me/hijackedjournalcheckerbot. It can answer questions about hijacked journals with acceptable reliability; however, as this is a trial version and for test purpose not wide spread usage, researchers should be cautious with its use. The aim was to show it is possible to have a research specific chatbot. This chatbot has the ability to answer questions pertaining to the evaluation of particular journals, the nature of hijacked journals, and various methods of detection. Furthermore, it can be useful in teaching novice researchers about the prevalence and dangers of hijacked journals. Figure 3 illustrates screenshots from chatbot. Questions that ChatGPT answered inaccurately were answered correctly by the Journal Checker Chatbot.

## Conclusion

 The current study analyzes the reliability of ChatGPT for journal quality evaluation. The results indicate that ChatGPT is not a reliable tool because it recommends questionable journals for publishing. As questionable journals are proliferating and their influence is spreading via a variety of means (e.g., emails, advertisements in search engines), a usable and accessible tool for both journal detection and related education is much needed. To show how it can meet this need, for test purpose, a chatbot entitled “Journal Checker Chatbot” was developed. This chatbot is a beta version and for test purpose that will undergo improvements to ensure an even higher level of accuracy. With future investment in its infrastructure, we will use predatory journals data to train this chatbot. The author will utilize advanced data technologies to gather lists of hijacked journals which the chatbot will use automatically to increase its accuracy and undermine the damage being caused by hijacked and predatory journals.

## Competing Interests

 No conflict of interest.

## Ethical Approval

 Not applicable.

## Funding

 There is no funding.
